# Immune organoids for Africa: a roadmap for bridging the innovation gap in infectious diseases and cancer research

**DOI:** 10.1186/s43046-026-00385-4

**Published:** 2026-07-15

**Authors:** Ahmad Onikoko Abdulgafar, Adeyemi Sallam Febilola, Catherine Rono, Chinemerem Henry Ugo, Saerimam Nzunde Markus, Mariam Iyabo Adeoba, Dagunduro Emmanuel Tolu

**Affiliations:** 1 Usmanu Danfodiyo University Sokoto, Sokoto, Nigeria; 2 Roswell Park Comprehensive Cancer Center, Buffalo, USA; 3 University of Nigeria Nsukka, Enugu, Nigeria; 4 University of Jos, Jos, Nigeria; 5 University of South Africa, Florida, Johannesburg, South Africa; 6 University of Lagos, Lagos, Nigeria

**Keywords:** Immune organoids, Biomedical sovereignty, Genomic sovereignty, Frugal bioengineering, Precision oncology, Global health equity, Sub-Saharan Africa

## Abstract

**Background:**

Sub-Saharan Africa bears approximately 25% of the global disability-adjusted life-year (DALY) burden yet contributes fewer than 3% of global health research outputs. HIV/AIDS, tuberculosis (TB), and malaria coexist with a rising cancer burden, with attributable mortality projected to increase 75% by 2050. Fewer than 0.2% of genome-wide association study (GWAS) participants are of African ancestry, constraining precision medicine relevance. Conventional two-dimensional cultures and murine models inadequately replicate immune dynamics, tissue-level pathogenesis, and African pharmacogenomics. Immune organoids (three-dimensional microphysiological constructs) offer human-relevant platforms for mechanistic immunology, drug screening, vaccine evaluation, and precision oncology.

**Method:**

A narrative review was conducted across PubMed, Scopus, Web of Science, and Google Scholar (January 2019–June 2025), integrating technical descriptors (lymph node-on-a-chip, patient-derived organoids, microphysiological systems) with regional keywords (sub-Saharan Africa, genomic sovereignty, STISA-2034, frugal bioengineering). Gray literature from WHO, World Bank, and Africa CDC was incorporated. Thematic synthesis identified technological advances, infrastructural barriers, regulatory gaps, and sociopolitical determinants of adoption.

**Result:**

Lymph node-on-a-chip, tonsil-derived, and tumor–immune co-culture organoids enable high-fidelity interrogation of host–pathogen and tumor–immune interactions. Adoption is constrained by infrastructure deficits, reagent costs, energy instability, bioinformatics gaps, and nascent regulatory frameworks. Scalable solutions include a three-tier hub framework (Coordinator–Generator–Collaborator), ABCOMICS for continental genomic data governance, frugal LCD-based bioprinting, and passive microfluidic perfusion. Harmonized ethical guidelines and community-engaged consent are essential for genomic sovereignty.

**Conclusion:**

A phased, STISA-2034-aligned roadmap integrating frugal bioengineering, ethical governance, and targeted capacity-building can position Africa as a global leader in human-centric biomedical research, accelerating precision medicine, pandemic preparedness, and health equity.

## Introduction

**Fig. 1:**
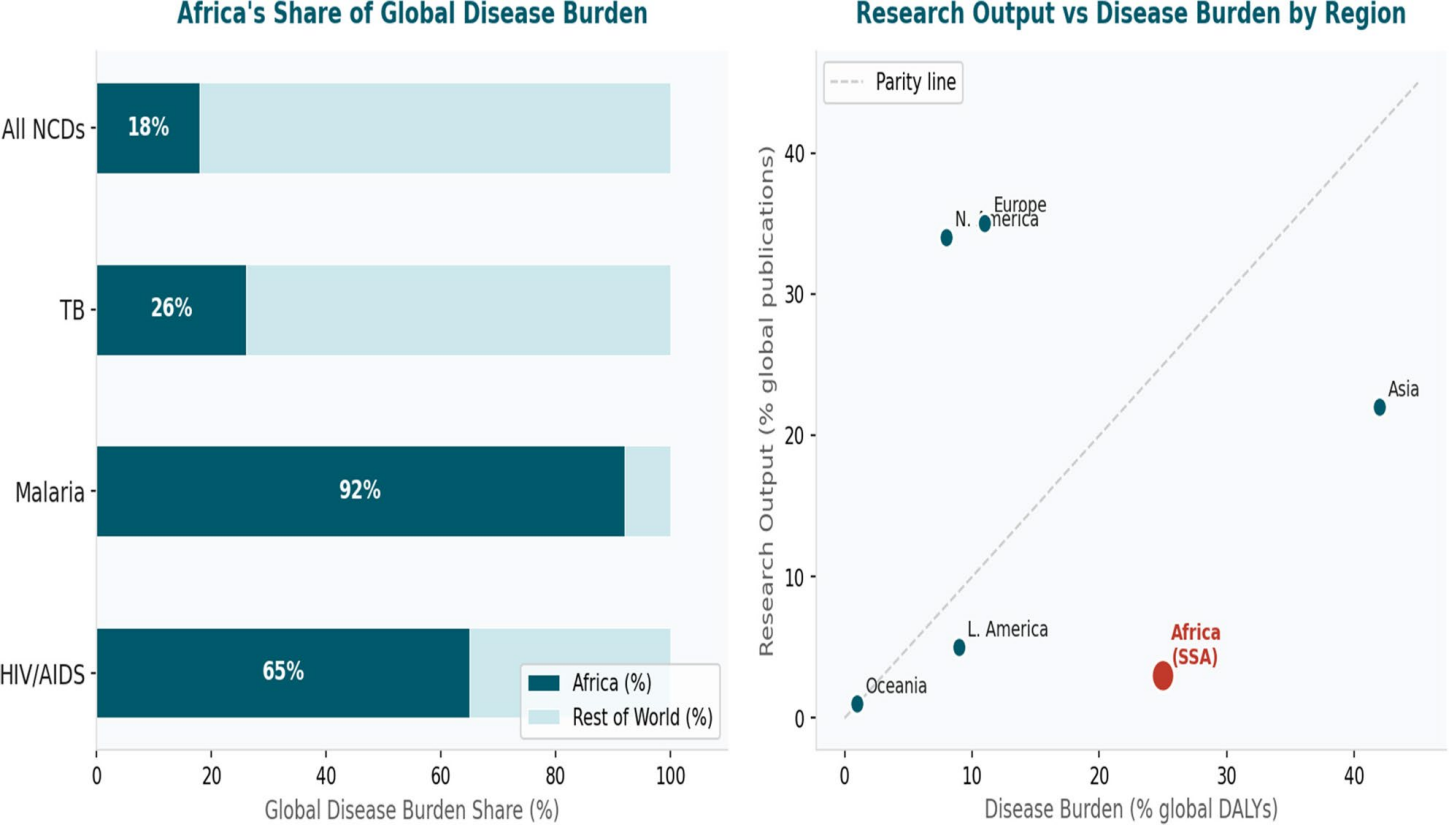
Africa’s disease burden relative to global research output. Left panel: Africa’s proportional share of global DALYs for HIV/AIDS, malaria, TB, and non-communicable diseases. Right panel: scatter plot showing the inverse relationship between disease burden and research output across global regions, illustrating the research equity gap. Data derived from the Global Burden of Disease Study 2021 [1] and the Global Cancer Observatory [23]

Sub-Saharan Africa faces an entrenched and widening gap between its disease burden and its biomedical research capacity. Despite global progress in reducing age-standardized mortality, average life expectancy in the region remains approximately 62 years, nearly two decades below the global frontier [1–3] Endemic infectious diseases, including HIV/AIDS, tuberculosis (TB), and malaria, continue to drive substantial morbidity and premature mortality, while non-communicable diseases, particularly cancers, are rising with striking speed (Fig. 1): cancer-attributable mortality is projected to increase by 75% before 2050 [2, 4, 5].

This dual burden is compounded by a fundamental limitation of contemporary preclinical research. Murine animal models fail to reproduce the immunological and tissue-level complexity of human disease due to species-specific differences in immune architecture, cytokine signaling, granuloma biology, and pathogen tropism [6, 7]. This translational gap contributes to the high attrition of therapeutic and vaccine candidates that succeed preclinically but fail in clinical trials. Simultaneously, fewer than 0.2% of participants in global GWAS studies are of African ancestry [8–10], severely limiting the relevance of precision medicine for populations with the world’s greatest genetic diversity [11, 12].

The structural origins of this crisis extend beyond scientific methodology. Africa’s biomedical innovation gap reflects historical reliance on extractive research models in which biological samples and intellectual outputs were generated locally but analyzed, published, and commercialized abroad with minimal local benefit [13, 14]. Prior initiatives, such as the H3Africa Consortium and the Africa CDC Pathogen Genomics Initiative, have advanced sequencing and bioinformatics capacity but remain bottlenecked by scalability, cost, and genomic sovereignty concerns [15, 16].

Immune organoids, a self-organizing, three-dimensional tissue construct derived from human pluripotent stem cells (hPSCs) or primary tissue-resident progenitors, represent a transformative response to these converging challenges [17–19] By faithfully recapitulating the cellular architecture, spatial organization, and functional dynamics of lymphoid organs, immune organoids enable controlled interrogation of host–pathogen interactions, tumor–immune crosstalk, and pharmacogenomic variability within a human tissue framework [18–20]. Their compatibility with patient-derived cells makes them uniquely suited to capture the genetic diversity, comorbidity profiles, and immune conditioning characteristic of African populations [10, 11].

This review constructs an agenda-setting roadmap for immune organoid implementation across Africa. Aligned with the African Union’s Science, Technology and Innovation Strategy for Africa 2034 (STISA-2034) [21] and the global “100-Day Mission” for pandemic response [22], the roadmap advocates for decentralized R&D, equitable innovation, and a transition from Africa as a technology recipient to a global leader in biomedical science.

## Methodology

This narrative review employs an interdisciplinary thematic synthesis to develop a strategic roadmap for immune organoid integration in Africa’s biomedical research landscape. Literature searches were conducted across PubMed, Scopus, Web of Science, and Google Scholar, restricted to January 2019 through June 2025. This window captures the maturation of 3D bioprinting and microfluidic organoid technologies and the post-COVID-19 recalibration of global pandemic preparedness. A cutoff of June 2025 was applied to preserve consistency between the qualitative thematic coding and the GBD 2023 quantitative triangulation; sources published thereafter were reviewed and incorporated selectively only where they materially reinforced conclusions already established within the primary search window.

Search strings combined technical descriptors—“immune organoids,” “lymph node-on-a-chip,” “patient-derived organoids,” “microphysiological systems,” “tumor–immune co-culture”—with strategic terms: “sub-Saharan Africa,” “genomic sovereignty,” “frugal bioengineering,” and “STISA-2034.” Inclusion criteria prioritized: (1) high-impact primary studies and reviews of immune organoid engineering and validation; (2) authoritative reports from WHO, Africa CDC, World Bank, and the GBD 2023 study on disease burden and research infrastructure; and (3) peer-reviewed ethical and regulatory analyses for African jurisdictions.

A problem-based narrative synthesis framework was employed rather than a PRISMA-compliant systematic review, enabling critical integration of interdisciplinary evidence spanning molecular biology, bioengineering, health economics, and political science. Quantitative indicators from GBD 2023 were triangulated with qualitative insights from innovation scholars to ensure scientific rigor and policy relevance.

## Immune organoids: concepts, types, and technological evolution

### Conceptual foundation

Immune organoids are three-dimensional, self-organizing cellular assemblies that recapitulate the structural and functional complexity of primary and secondary lymphoid organs such as the thymus, lymph nodes, spleen, and tonsils, in vitro [16, 17]. Their conceptual origins lie in the recognition that two-dimensional monolayer cultures fundamentally misrepresent tissue physiology by eliminating cell polarity, spatial signaling gradients, and matrix-mediated mechanical cues that govern immune cell differentiation, migration, and activation [18, 24]. Murine models, while more physiologically representative, are constrained by differences in immune gene expression, cytokine repertoires, granuloma formation, and pathogen susceptibility [6, 7].

The transformative shift to contemporary organoid systems emerged from the convergence of three enabling technologies: advances in hPSC biology [24, 25], biomaterials engineering capable of presenting physiologically relevant extracellular matrix signals [26], and microfabrication platforms enabling precise spatial patterning of cellular components [25, 27]. Immune organoids are generated from either hPSCs, offering scalability, genetic manipulability, and standardization, or primary tissue-resident progenitors from tonsils, lymph nodes, or spleen, which preserve donor-specific immune repertoires and epigenetic signatures critical for translational and population-specific immunology [24, 28, 29].

### Classification of immune organoid platforms

**Fig. 2:**
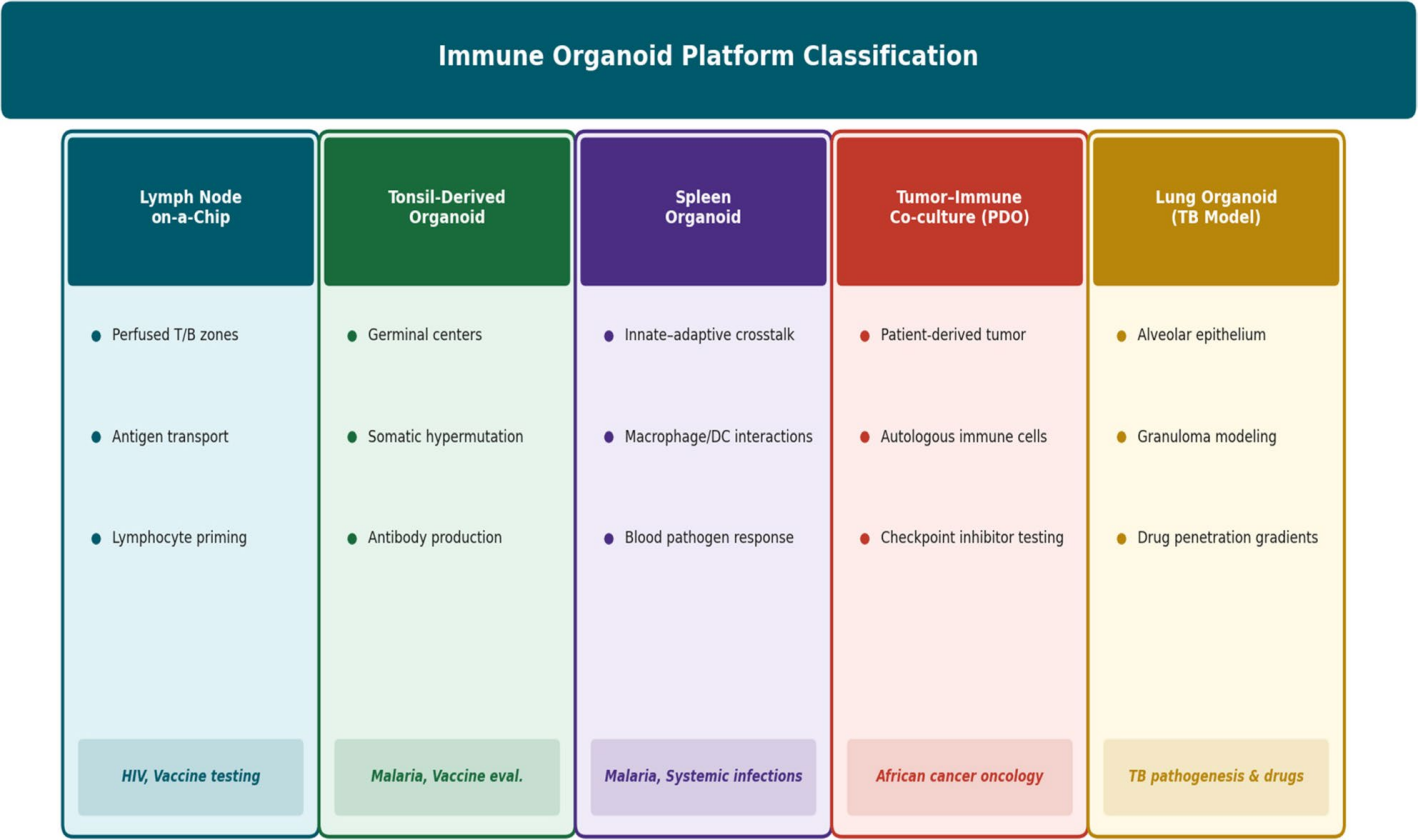
Classification and functional architecture of primary immune organoid platforms. Five principal systems are depicted with key cellular components, features, and African disease relevance. The spectrum ranges from static multicellular aggregates to perfused, bioprinted microphysiological systems incorporating stromal and endothelial components. Compiled from Li et al. (2025) [18], Olawade et al. (2025) [20], and Wagar et al. (2021) [24]

Table 1; Fig. 2 provide a comparative overview of contemporary immune organoid platforms and their relevance to African disease priorities.

**Table 1:** Comparative overview of immune organoid platforms, key functional characteristics, and relevance to African disease contexts

Platform	Source	Key Features	African Disease Relevance	Limitation
Lymph node-on-a-chip [24, 27, 28]	Primary / hPSC	Perfused T/B zones; antigen transport; lymphocyte priming under flow	HIV latency; vaccine response testing	Complex microfluidic fabrication
Tonsil-derived organoid [24, 30, 31]	Surgical tissue	Germinal centers; somatic hypermutation; antigen-specific antibody production	Malaria immunity; vaccine immunogenicity evaluation	Donor variability; supply constraints
Spleen organoid [20, 30]	iPSC / splenic cells	Innate–adaptive crosstalk; macrophage–DC interactions	Malaria; blood-borne infections	Limited vascularization
Tumor–immune co-culture (PDO) [32, 33]	Primary tumor biopsy	Autologous immune cells; checkpoint inhibitor testing; CAR-T evaluation	African-specific cancer subtypes; pharmacogenomics	High cost; requires biobanking infrastructure
Lung organoid (TB model) [34, 35]	hPSC / lung progenitors	Alveolar epithelium; granuloma modeling; drug penetration gradients	TB pathogenesis; drug efficacy testing	Lacks complete adaptive immunity

*hPSC* human pluripotent stem cell, *iPSC *induced pluripotent stem cell, *PDO *Patient-derived organoid

### Enabling technologies

Three-dimensional bioprinting has been central to advancing organoid spatial fidelity [26]. Precise deposition of immune, stromal, and endothelial cells within biomimetic hydrogels facilitates reproducible reconstruction of lymphoid tissue architecture [26, 28]. Sacrificial bioprinting strategies permit the incorporation of perfusable microchannels that emulate lymphatic and vascular conduits, enabling sustained nutrient exchange, cytokine gradients, and immune cell trafficking, overcoming diffusion limitations of static organoids [27, 29]. Recent 4D bioprinting extensions and organoid fusion strategies allow modeling of systemic immune interactions across tissue compartments [26, 28, 29].

Fibroblastic reticular cells (FRCs), lymphatic endothelial cells, and blood endothelial cells form the structural backbone of lymphoid organs in vivo, guiding immune cell positioning through chemokine gradients and extracellular matrix cues [24, 27, 28]. Their integration into organoid systems provides the stromal framework necessary for physiologically faithful immune behavior, a critical advance over early multicellular aggregates [16, 17].

## Relevance to infectious disease research in Africa

### Tuberculosis

Granuloma Modeling and Drug Evaluation Tuberculosis illustrates both the limitations of animal models and the promise of immune organoids [25, 34, 35]. Human Mycobacterium tuberculosis infection generates necrotic, hypoxic granulomas with heterogeneous immune cell distribution and uneven antibiotic penetration, features poorly reproduced in mice.^35^ The immunological divergence between human and murine granuloma biology has contributed directly to the poor predictive validity of murine TB preclinical data [6, 15, 35].

Human lung organoids derived from hPSCs capture alveolar epithelial architecture and support infection by virulent Mtb H37Rv within a three-dimensional tissue context [17, 27]. These systems maintain structural and transcriptional stability across passages, enabling longitudinal analysis of infection dynamics and host immune activation [17]. Organoid-based evaluation of frontline agents, including rifampicin and bedaquiline, demonstrates physiologically relevant dose-response profiles shaped by oxygen gradients and localized infection foci that static cultures cannot replicate [25, 34]. In African contexts, where TB coexists with HIV (which profoundly reshapes granuloma immunology) [35] and malnutrition, organoid systems can incorporate these comorbid immune perturbations for more accurate therapeutic prediction [34, 35].

### Malaria: germinal center dynamics and vaccine immunogenicity

**Fig. 3:**
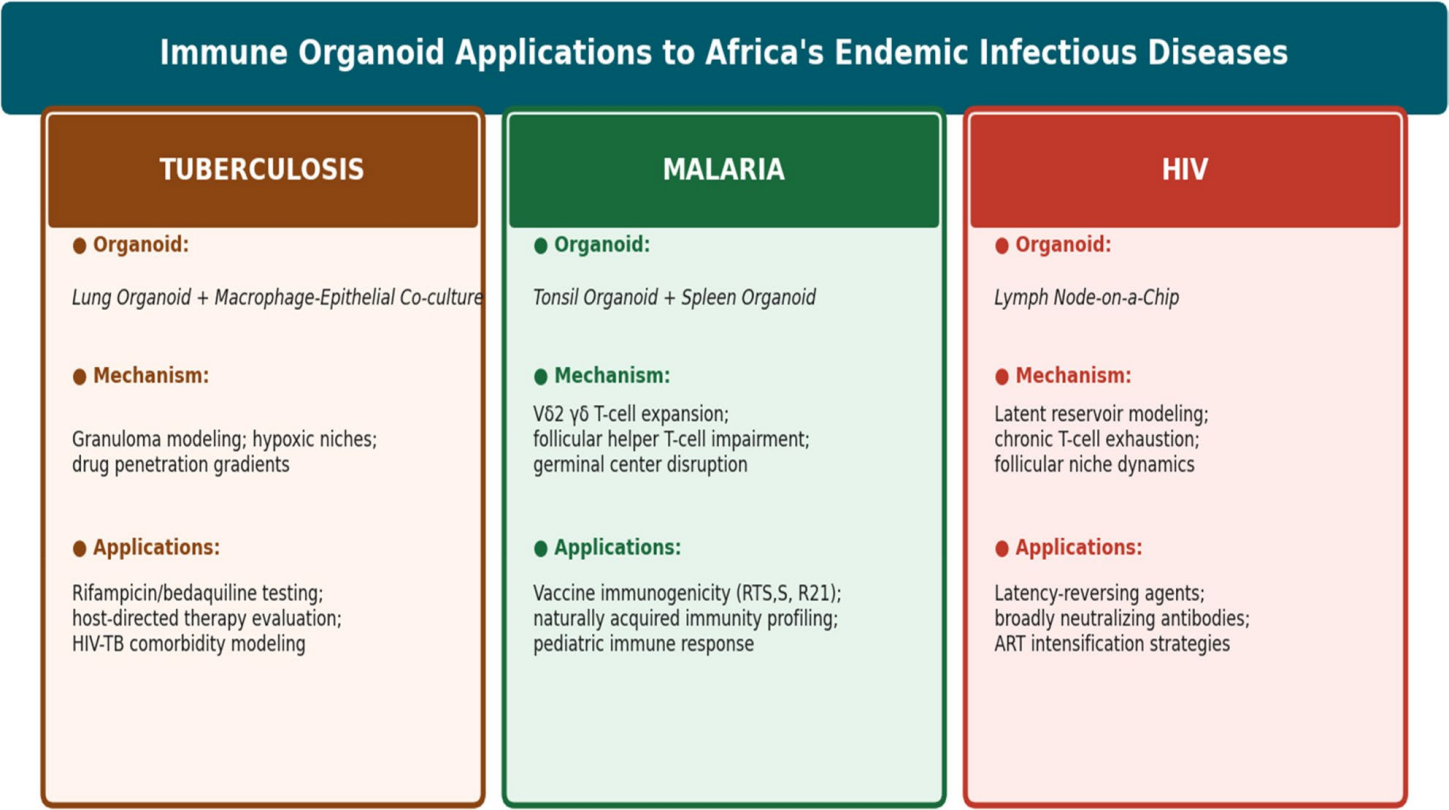
Application of immune organoids to Africa’s three major endemic infectious diseases. Each column maps the relevant organoid platform, immunopathological mechanism, and translational application for tuberculosis, malaria, and HIV, respectively. Data synthesized from Mbonye et al. (2023) [36] and Wagar et al. (2021) [24]

Malaria remains the leading infectious killer of children in sub-Saharan Africa, yet the immunological basis of naturally acquired immunity and suboptimal vaccine performance remain incompletely understood [30, 31]. Tonsil and spleen immune organoids have provided key mechanistic insights: exposure to Plasmodium falciparum-infected erythrocytes drives clonal expansion of cytotoxic Vδ2 γδ T cells [30]. Critically, this expansion occurs at the expense of follicular helper T-cell generation, impairing germinal center reactions and constraining antigen-driven antibody maturation, a plausible mechanism underlying limited durability of malaria-specific humoral immunity (Fig. 3) [22, 24, 31].

Validation studies using immune tissues from Ugandan children confirmed that organoid findings directly recapitulate physiologically relevant dynamics in endemic populations [37] These correspondence positions tonsil organoids as indispensable tools for iterative malaria vaccine candidate screening [24, 31] and for assessing germinal center formation, antibody affinity maturation, and memory B-cell generation in donor-matched systems [24].

### HIV: reservoir modeling and latency research

HIV cure research confronts a fundamental anatomical challenge: the virus establishes latent reservoirs predominantly within CD4 + T cells residing in lymphoid follicular and parafollicular zones, the precise microenvironments that lymph node-on-a-chip systems recreate [36, 38]. These platforms support sustained HIV infection and controlled analysis of viral dissemination, latency establishment, chronic inflammation, and T-cell exhaustion [28, 36, 38].

In African contexts, co-infections with TB, cytomegalovirus, and other chronic pathogens amplify immune activation and CD4 + T-cell turnover, influencing reservoir seeding [30, 36] Lymphoid organoids provide an experimentally tractable system for investigating how these comorbid immune states affect latency-reversing agent efficacy and immunomodulatory interventions such as broadly neutralizing antibody combinations (Fig. 3) [28, 36].

### Emerging and re-emerging viral threats: viral hemorrhagic fevers and COVID-19

Africa’s infectious disease burden extends beyond HIV, TB, and malaria to recurrent outbreaks of viral hemorrhagic fevers (Ebola, Marburg, Lassa) and the ongoing legacy of COVID-19, both of which expose the same biosafety and animal-model constraints motivating this review. Lung air–liquid-interface organoids that preserve endogenous tissue-resident immune populations (T, B, NK, myeloid) have demonstrated robust, donor-specific SARS-CoV-2 T-cell responses following infection, offering a platform for regional vaccine and therapeutic evaluation independent of imported cohort data [39]. For viral hemorrhagic fever pathogens requiring BSL-4 containment, purified stem-cell-derived hepatocyte systems have revealed that Ebola and Lassa viruses, despite both causing hemorrhagic fever clinically, produce markedly different transcriptional and cytopathic effects on human liver cells [40]. Such platforms, generated and validated outside BSL-4 facilities and infected only under appropriately contained conditions, offer African laboratories a route to mechanistic VHF data that would otherwise be entirely inaccessible. Integrating VHF- and COVID-19-relevant organoid platforms into the roadmap’s Tier 2/3 hub structure (Sect.  9) would extend its scope beyond the three endemic diseases currently depicted in Fig. 3.

## Applications in cancer immunology and precision oncology

### Patient-derived tumor organoids and African cancer biology

Cancer has emerged as a public health emergency in Africa, with mortality-to-incidence ratios markedly higher than those in high-income regions [2, 41]. Late diagnosis, restricted access to targeted therapies, and the systematic underrepresentation of African tumor biology in global oncological datasets compound this burden [2, 41, 42]. Patient-derived tumor organoids (PDOs), established directly from primary tumor biopsies or surgical specimens, preserve histological architecture, genomic landscape, epigenetic states, and intra-tumoral heterogeneity of the parental tumor [32, 43, 44], making them uniquely suited for individualized drug testing, resistance profiling, and biomarker discovery [35, 44].

In African contexts, where tumor subtypes frequently differ biologically from those characterized in European and North American cohorts, including higher rates of triple-negative breast cancer, infection-associated cervical malignancies, and Helicobacter pylori-driven gastric cancer [2, 41], PDOs provide a mechanism for interrogating cancer biology within a population-relevant molecular framework [44, 45].

### Pharmacogenomics and precision drug testing

African populations harbor the world’s greatest genetic diversity, including functionally significant polymorphisms in genes governing drug metabolism, DNA repair, and immune regulation [10, 11]. Cytochrome P450 variants, particularly CYP2D6 and CYP2B6, occur at higher frequencies and with distinct allelic distributions across African ethnic groups, substantially altering the metabolic activation and clearance of anticancer agents, including tamoxifen, cyclophosphamide, and imatinib [11].

PDO-based functional pharmacogenetic assays enable direct empirical assessment of drug sensitivity within biologically faithful tumor models derived from African patients, circumventing the limitations of genotype-based prediction algorithms calibrated on non-African populations [44, 46, 47]. For example, PDO assays can directly quantify impaired tamoxifen-to-endoxifen conversion in breast cancer patients with poor-metabolizer CYP2D6 genotypes, providing actionable therapeutic guidance that genotyping alone cannot deliver [37, 47].

### Tumor–immune co-culture systems and immunotherapy prediction

Immunocompetent tumor organoids, PDOs co-cultured with autologous tumor-infiltrating lymphocytes, peripheral blood mononuclear cells, or engineered CAR-T cells, enable dynamic assessment of immune surveillance, T-cell infiltration, cytotoxic killing, and immune checkpoint blockade resistance within a patient-specific tumor microenvironment [33]. These platforms are critical in African settings where checkpoint inhibitors such as pembrolizumab and nivolumab are increasingly entering oncological practice, yet predictive biomarkers validated in African tumor–immune contexts remain absent [33, 46].

Endemic co-infections in Africa, including HIV, hepatitis B and C, and helminth infections, profoundly alter the immunological milieu within which tumors arise and respond to therapy [5, 15]. These factors condition immune exhaustion, CD4 + T-cell depletion, and type-2 cytokine skewing in ways not captured by standard trial populations [5, 33]. Immunocompetent tumor organoids incorporating African donor cells permit controlled experimentation within this complex immune landscape, generating Africa-specific biomarker hypotheses for prospective clinical validation [33].

At the institutional level, the Nigerian Institute of Medical Research (NIMR) and the Africa Health Research Institute (AHRI) are developing tumor organoid biobanks reflecting regional cancer burdens [12, 13]. These shared platforms serve as the foundation for collaborative drug screening, biomarker validation, and translational oncology research anchored in African biological realities [12, 13, 33].

## Infrastructure, capacity, and workforce requirements

### From extractive models to regional scientific sovereignty

Sustainable adoption of immune organoid technologies demands a structural transition away from extractive “vampire” or “mosquito” research models [9, 12] toward regional scientific sovereignty encompassing local control over biological samples, data governance, analytical pipelines, and downstream innovation. Without this foundation, immune organoid research risks reproducing historical inequities in which African tissues generate discoveries benefiting primarily external institutions [12, 48].

### The three-tier hub framework

Table 2 describes the three-tier national and regional institutional framework providing a pragmatic architecture for biomedical sovereignty at the continental scale.

**Table 2:** Three-tier institutional framework for immune organoid research governance and implementation in Africa

Tier	Role	Core Functions	Example Institutions
Tier 1 Coordinator	Strategic oversight	Policy alignment; ethical governance; regulatory harmonization; international partnership management; national priority-setting	Africa CDC; AU Commission; National Health Ministries; AMRH Secretariat
Tier 2 Generator	Technical production	Organoid fabrication; bioprinting; microfluidics; advanced imaging; biobanking with clinical metadata linkage; protocol development and validation	KEMRI (Kenya); AHRI (South Africa); NIMR (Nigeria); Uganda Virus Research Institute
Tier 3 Collaborator	Clinical application	Drug testing; clinical validation; implementation research; community outreach; training delivery; protocol application	Teaching hospitals; regional laboratories; district health institutes; public health programs

Framework adapted from Beyene et al. (2021) [12], Africa CDC Strategic Plan 2023–2027 [13]

*KEMRI* Kenya Medical Research Institute, *AHRI *Africa Health Research Institute, *NIMR *Nigerian Institute of Medical Research, *AMRH *African Medicines Regulatory Harmonization

### Enabling infrastructure layers

Four enabling infrastructure layers must be developed in parallel. In genomics and multi-omics, ABCOMICS and CESORA exemplify the transition toward local governance of next-generation sequencing data. In biobanking, platforms including ALTBio provide ethically governed repositories of immune tissues linked to rich clinical metadata, essential for diverse, well-annotated organoid models [49]. In data science, H3ABioNet and NIH-supported hubs are building distributed computational capacity, with a target of training at least 10,000 data-literate biomedical scientists [12, 49]. In regulatory science, the AMRH initiative aligns standards across jurisdictions to facilitate multicountry clinical translation [50].

Workforce development must accompany infrastructure investment. Immune organoid research requires scientists capable of integrating stem cell biology, bioengineering, single-cell genomics, and clinical immunology, expertise not produced by conventional siloed training [12]. Recent investments, including approximately 58 million USD from NIH and complementary Pandemic Fund allocations, are catalyzing interdisciplinary graduate programs, short-course intensives at Generator Hubs, and embedded research fellowships [12, 13, 22]. Sustained political commitment from African governments remains indispensable [9, 12, 23, 48] makes robust oversight a moral and scientific imperative. Organoids compound these concerns: a single tissue donation can generate organoid lines passaged, expanded, and genetically modified for years, with implications for donor privacy, benefit sharing, and institutional intellectual property that original consent processes did not anticipate [51].

Regulatory frameworks across Africa are evolving unevenly. Nigeria’s 2025 National Biotechnology Policy review represents a pivotal opportunity to integrate organoid systems, hPSC research, and gene-editing explicitly into national governance [49, 50, 52]. Similar modernization processes are underway in Kenya, Rwanda, and South Africa [50, 53, 54]. Most countries still lack explicit definitional frameworks for organoids within existing regulatory categories, creating ambiguity that simultaneously impedes ethical innovation and opens permissive gaps [49, 53, 55].

### Culturally responsive consent models

Standard written consent models, imported from high-income settings, may be structurally inaccessible in communities characterized by linguistic diversity, variable literacy, and culturally grounded conceptions of bodily ownership diverging from Western bioethical frameworks [4, 48]. For immune organoid research, where tissues may be expanded, genetically manipulated, and used in future unspecified studies, one-time transactional consent is ethically inadequate [20, 48].

Layered consent frameworks allowing participants to specify acceptable uses of their materials, community engagement processes preceding individual sample collection, and verbal consent procedures with independent witnesses have demonstrated superior comprehension, legitimacy, and retention in low-resource settings. These approaches should be institutionalized within ethics review processes, paired with ongoing donor communication and accessible benefit-sharing mechanisms [48, 53, 55].

### Sociocultural acceptability and regulatory harmonization

In North and West Africa, Islamic bioethical principles shape public attitudes toward stem cell technologies. The 2003 scholarly Fatwa permitting stem cell research conducted with adult tissues and ethically obtained materials [56] provides a legitimizing foundation for immune organoid work in these regions, as contemporary platforms rely primarily on adult stem cells or reprogrammed somatic cells rather than embryonic sources [56]. Proactive engagement with religious scholars and community leaders can facilitate social license and align scientific practice with local moral frameworks [56].

Data governance is equally critical. Immune organoid research generates commercially valuable datasets, whole-genome sequencing, single-cell transcriptomics, high-content imaging, requiring enforceable mechanisms ensuring African institutional governance, regional data processing, and equitable benefit-sharing agreements [48]. The ABCOMICS framework and the African Union’s Model Law on Bioethics provide nascent but important foundations. South Africa’s National Health Act offers one of the continent’s most mature regulatory environments for advanced biomedical research, with explicit guidance on human tissue use, biobanking, and benefit sharing [48, 53, 55].

## Barriers to adoption and frugal mitigation strategies

Table 3 provides a structured barrier–mitigation mapping. Each major structural obstacle to immune organoid adoption is paired with contextually adapted frugal engineering and institutional responses.

**Table 3:** Principal barriers to immune organoid adoption in Africa and evidence-based mitigation strategies

Barrier	Description	Mitigation Strategy
High hardware cost	Commercial bioprinters: $15,000–$30,000; beyond most laboratory budgets	LCD-resin printers ($150–$600) repurposed for microfluidic mold fabrication; open-source CAD designs; consortium purchasing arrangements
Reagent dependency	Proprietary extracellular matrices (e.g., Matrigel) subject to cold-chain failures, import delays, and currency volatility	Local GelMA, alginate, and fibrin-based matrix synthesis; dental-grade biocompatible resins; regional reagent hubs with bulk import capacity
Energy instability	Continuous perfusion, CO₂ incubation, and imaging systems require uninterrupted power	Passive capillary-driven and gravity-based microfluidic perfusion; solar-backed UPS systems for critical equipment; passive incubation designs
Bioinformatics deficit	Multi-omics datasets exceed local analytical capacity; reliance on foreign cloud infrastructure	ABCOMICS continental governance framework; H3ABioNet expansion; regional HPC nodes; targeted training of ≥ 10,000 data scientists
Nascent regulation	Organoids lack explicit classification in most African regulatory frameworks	Anticipatory regulation through AMRH; national policy reviews (e.g., Nigeria 2025 Biotech Policy); WHO draft organoid quality guidelines
Workforce gaps	Multidisciplinary expertise in stem cell biology, bioengineering, and bioinformatics largely absent	Interdisciplinary PhD curricula; short-course intensives at Generator Hubs; NIH + Pandemic Fund embedded fellowships
Ethical and consent complexity	Standard consent models inaccessible in low-literacy or multilingual populations; organoid-specific consent insufficiently regulated	Layered consent frameworks; community engagement protocols; verbal consent with independent witnesses; institutionalized ethics review integration

Sources: Beyene et al. (2021) [12]; Mulder et al. (2021) [49]; WHO (2024) [51]

*CAD* Computer-aided design, *GelMA *Gelatin methacryloyl, *UPS* Uninterruptible power supply, *ABCOMICS *African Bioinformatics and Computational Omics Infrastructure, *AMRH *African Medicines Regulatory Harmonization

### Frugal bioengineering: hardware and reagent solutions

Hardware cost constitutes the most visible entry barrier. Commercial 3D bioprinters used in high-income settings cost $15,000–$30,000, placing them beyond most African laboratory budgets [57]. LCD-based resin printers, widely available at $150–$600, can be configured to fabricate high-resolution microfluidic molds and perfusion chip architectures sufficient for immune organoid culture applications [57, 58]. When combined with open-source design files and locally sourced components, these systems achieve sufficient precision for lymphoid and tumor–immune organoid applications [58, 59].

**Fig. 4:**
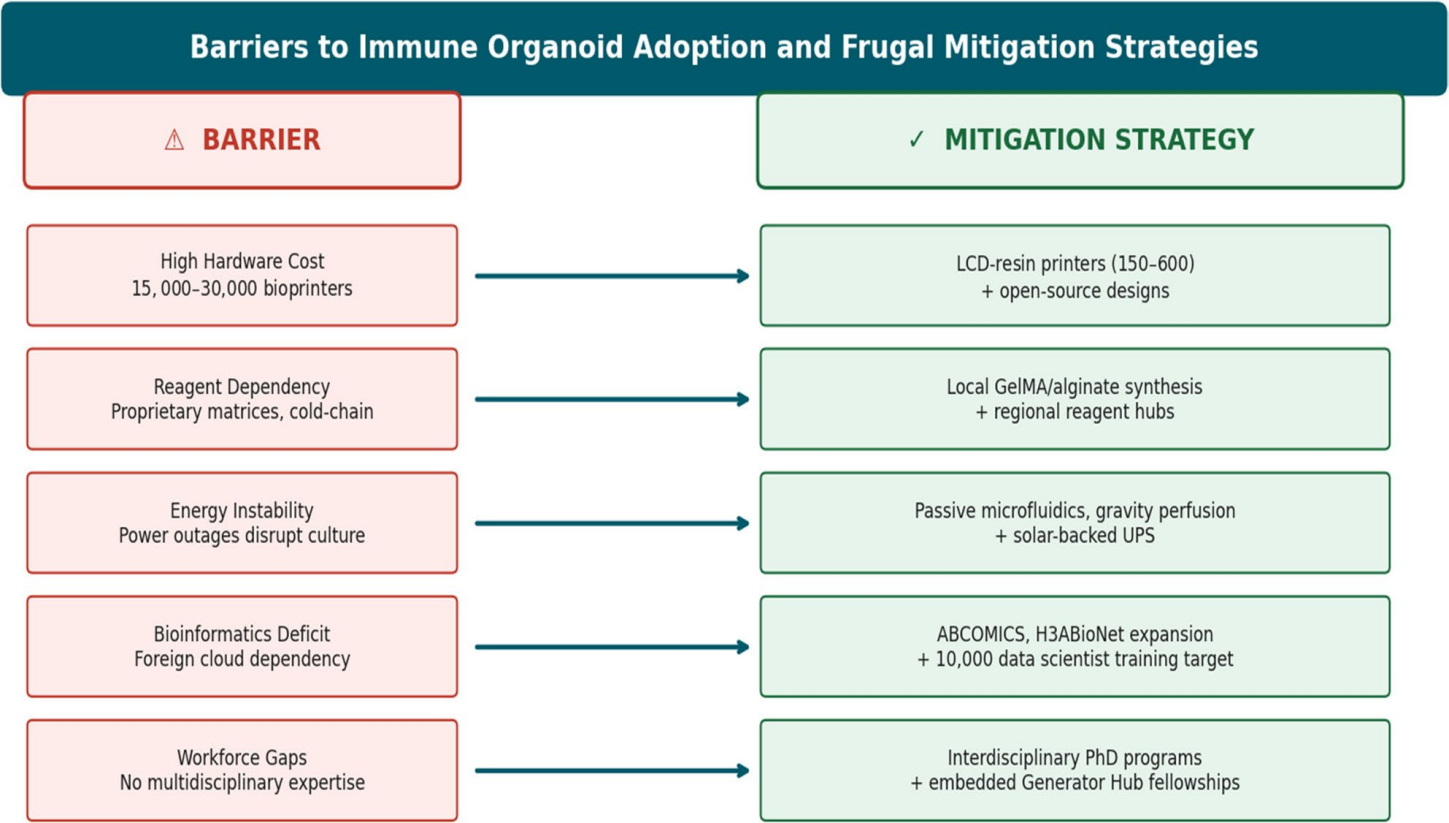
Barrier-to-solution mapping for immune organoid implementation in Africa. Each structural obstacle is linked to a contextually adapted frugal engineering or institutional mitigation strategy. Compiled from Beyene et al. (2021) [12]

Reagent dependency compounds the challenge (Fig. 4). Locally synthesized hydrogel alternatives, including alginate, gelatin methacryloyl (GelMA), and fibrin-based matrices functionalized with adhesion peptides and growth factor binding domains, can approximate commercial matrices at substantially lower cost with domestically manageable supply chains [59, 60]. Iterative contextual validation against established organoid performance benchmarks is achievable within Generator Hub infrastructure [60, 61].

### Energy-independent perfusion and AI-powered living labs

Passive microfluidic solutions, including capillary-driven flow systems, gravity-based perfusion chambers, and evaporation-driven microchannels, decouple organoid viability from uninterrupted electricity, enabling sustained nutrient exchange and immune cell trafficking across power interruption events.^62^ Complementary solar-backed UPS systems sized for critical incubators, biosafety cabinets, and imaging platforms provide practical interim resilience (Fig. 4) [62, 63].

Collaborative “AI-powered Living Labs”, developed through partnerships between institutions such as Stanford’s Prakash Lab and African universities, integrate low-cost hardware, machine learning-assisted quality control, and participatory co-design [63, 64]. These environments prioritize co-creation of diagnostics, bioreactors, and organoid platforms with rather than for African research communities, ensuring contextually grounded technological development [62–64].

## Roadmap for implementation

The strategic deployment of immune organoid platforms requires a phased, STISA-2034-aligned implementation roadmap [63]. Table 4 provides a structured overview.

**Table 4:** Phased implementation roadmap for immune organoid platforms in Africa (2025–2031), aligned with STISA-2034 strategic priorities

Phase	Timeline	Key Activities	Expected Outcomes
Phase I Governance & Policy Validation	2025–2027	Establish national Immune Organoid Coordination Groups; regulatory scoping exercises; ethical framework validation; community trust-building; national priority disease identification aligned with burden profiles	National governance structures in ≥ 5 Member States; harmonized consent models; regulatory clarity; cross-sector stakeholder buy-in
Phase II Infrastructure Scaling & Consolidation	2027–2029	Designate ISO-certified Generator Hubs (e.g., KEMRI, AHRI); invest in frugal bioprinting and microfluidic platforms; integrate biobanking; workforce specialization programs; Tier 3 protocol distribution	≥ 3 operational regional hubs; validated frugal organoid platforms; trained interdisciplinary cohort of ≥ 500 scientists; shared organoid biobanks with ≥ 1,000 patient-derived specimens
Phase III Health Systems Integration	2029–2031	Embed organoid evidence in cancer control plans, AMR strategies, and vaccine deployment frameworks; regulatory uptake of organoid-generated data; public–private partnerships for local diagnostic and therapeutic development	Organoid-informed clinical guidelines in ≥ 3 disease areas; locally relevant diagnostics in development; Africa recognized as global contributor to organoid science

Roadmap developed from STISA-2034 strategic framework, Africa CDC Strategic Plan 2023–2027 [13]and Beyene et al. (2021) [12]

*KEMRI* Kenya Medical Research Institute, *AHRI *Africa Health Research Institute, *AMR *Antimicrobial resistance

### Phase I: governance and policy validation (2025–2027)

**Fig. 5:**
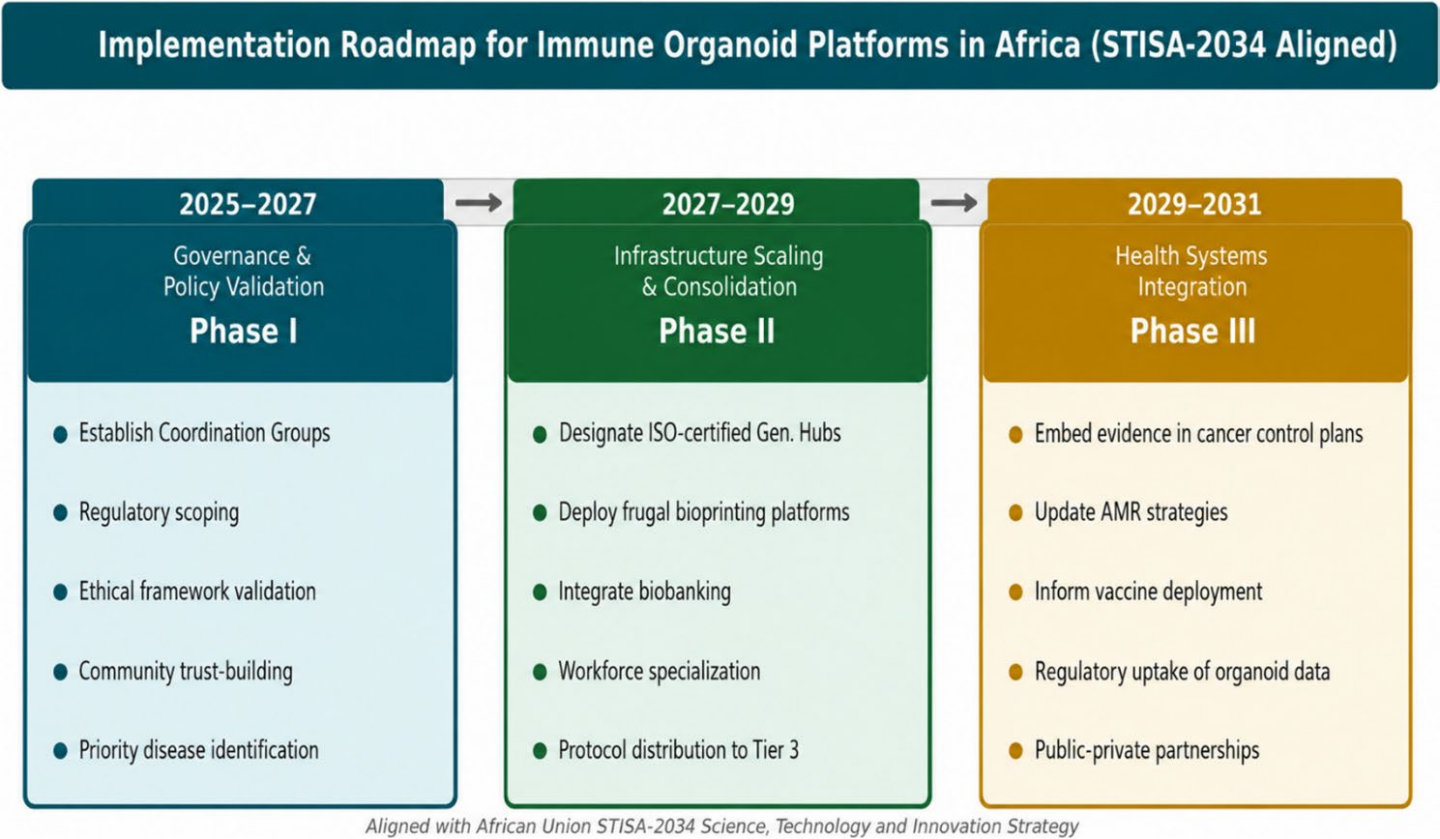
Three-phase implementation roadmap for immune organoid platforms in Africa (2025–2030). Each phase builds sequentially on governance, infrastructure, and translational integration milestones, with explicit alignment to STISA-2034 strategic priorities. Africa CDC Strategic Plan [13], and CEPI 100 Days Mission [22]

The inaugural phase (Fig. 5) prioritizes normative alignment and institutional trust-building over laboratory expansion [21]. Member States establish national Immune Organoid Coordination Groups comprising scientists, bioethicists, regulators, clinicians, community representatives, and policymakers. Regulatory scoping exercises classify immune organoids within existing biomedical and medicinal product frameworks, reducing ambiguity for investigators and funders [24]. Community trust-building activities, particularly in relation to tissue donation, data governance, and benefit sharing, are foundational to ensuring that downstream technological adoption is grounded in public legitimacy [24, 65].

### Phase II: infrastructure scaling (2027–2029)

The second phase (Fig. 5) concentrates advanced capabilities in strategically positioned Generator Hubs [12, 13] Centers such as KEMRI and AHRI are prime candidates for designation, with investments focused on modular bioprinting, passive and active microfluidics, biobanking with clinical metadata linkage, and workforce specialization [13, 49]. Generator Hubs carry an explicit mandate to transfer protocols, provide training, and distribute validated organoid constructs to surrounding Tier 3 institutions, ensuring that advanced capabilities diffuse rather than concentrate [12, 13].

### Phase III: health systems integration (2029–2031)

The third phase (Fig. 5) centers on translational integration: systematic incorporation of organoid-derived evidence into national health policy [21]. Cancer control plans incorporate organoid-informed biomarker panels and drug selection criteria [2, 46]. AMR strategies integrate organoid-generated pharmacodynamic data [7, 66]. Regulatory agencies develop standards for organoid data admissibility in product approval pathways [51]. Public–private partnerships channel organoid-based discoveries toward locally manufactured diagnostics and therapeutics [13, 15, 53].

## Implications for global health equity and pandemic preparedness

Regional scientific self-reliance is increasingly recognized as a prerequisite for global health security rather than its antithesis [22]. The COVID-19 pandemic exposed the structural vulnerability created when entire regions depend on external systems for pathogen characterization, vaccine testing, and therapeutic validation [22, 49]. Immune organoids directly address this vulnerability: within the “100-Day Mission” framework [22], pathogen-specific organoid challenges using mucosal respiratory or lymphoid systems can generate immunogenicity and safety signals within weeks of pathogen characterization, without requiring parallel animal model adaptation or overseas laboratory access [22, 43].

The systematic exclusion of African genetic and immunological diversity from early-stage biomedical research has measurable scientific and clinical consequences [8–10]. Therapeutics and vaccines optimized in populations of predominantly European ancestry can exhibit reduced efficacy or unanticipated toxicity in African populations with distinct drug metabolism profiles, HLA allele frequencies, and endemic pathogen-conditioned immune states [10, 11]. Immune organoids derived from African donors introduce this diversity at the discovery stage [8, 10], embedding population relevance as a foundational experimental condition rather than a post-hoc consideration [8–10].

Africa is a recognized hotspot for emerging and re-emerging zoonotic pathogens driven by ecological change, urbanization, and human–animal interfaces [7, 66]. Immune organoid models of respiratory, gastrointestinal, and lymphoid tissues allow African scientists to generate context-specific immunological data feeding directly into global surveillance and response networks [7, 22, 66]. This ensures that African populations become primary beneficiaries of early protective interventions rather than late recipients, operationalizing the principle that global health security begins with strong regional science [22, 66].

## Future research directions

Table 5 summarizes priority future research directions in immune organoid science and their specific relevance to African biomedical needs.

**Table 5:** Priority future research directions in immune organoid science and their specific relevance to African populations

Research Priority	Description	African Relevance
Multi-organ interaction platforms	Couple immune organoids with gut-, lung-, or liver-on-a-chip for ADME and systemic immune modeling	Comorbid infections; nutritional variation; polypharmacy and drug–drug interaction profiling
AI-driven fabrication optimization	Machine learning for batch standardization, culture condition optimization, and high-content image analysis	Reduce inter-laboratory variability across resource-limited settings; enable remote quality control
De novo immune memory engineering	Perturb homeostatic networks with sequential antigen challenges to observe in vitro memory state acquisition and maintenance	Vaccine durability; immune exhaustion under chronic infection; repeated endemic pathogen exposure
Spatial multi-omics integration	Apply GeoMx Digital Spatial Profiling and Visium spatial transcriptomics to resolve architecture–function relationships	Granuloma heterogeneity in TB; immune exclusion in African cancer subtypes; HIV reservoir topography
4D bioprinting and organoid fusion	Dynamically evolving constructs coupled with fused mucosal–lymph node organoid systems	Mucosal vaccine delivery route optimization; systemic immune–pathogen interaction modeling

Directions derived from Li et al. (2025) [18]

*ADME* Absorption, distribution, metabolism, excretion, *GeoMx* *DSP *GeoMx Digital Spatial Profiling

Multi-organ interaction platforms represent the most immediately actionable future direction [26]. Coupling immune organoids with gut-on-a-chip, lung-on-a-chip, or liver-on-a-chip systems within shared perfusion circuits enables simultaneous systemic drug pharmacokinetics and immune modulation modeling, which are critical for Africa’s polypharmacy and co-infection landscapes [26, 27].

Artificial intelligence will transform multiple levels of organoid research. Machine learning approaches can standardize organoid fabrication by detecting and correcting batch-to-batch variability in real time, essential for reproducible organoid generation across distributed African research networks [42]. Deep learning applied to high-content imaging, spatial transcriptomics, and single-cell proteomics will enable biomarker discovery at scales intractable by conventional analysis [42],

Spatial multi-omics, particularly GeoMx Digital Spatial Profiling [42], will allow investigators to resolve how immune function is architecturally constrained within organoid tissue, providing mechanistic insight into granuloma heterogeneity in TB, immune exclusion in African tumor microenvironments [33, 42], and HIV reservoir topography in lymphoid structures [36, 38].

## Conclusion

Immune organoids represent more than a methodological advance in preclinical research; they constitute a strategic medium through which Africa can attain genuine biomedical scientific sovereignty [15, 18, 20]. By enabling mechanistic, predictive, and interventional experimentation that leverages the continent’s unmatched genetic and immunological diversity, immune organoids facilitate a structural shift from descriptive epidemiology toward translational science, generating knowledge directly relevant to African populations [8–10, 21].

The scientific case for adoption is compelling across three domains: in infectious disease research, immune organoids resolve the critical translational deficit of animal models for TB, malaria, and HIV; [36] in precision oncology, PDOs enable population-relevant pharmacogenetic testing for cancer subtypes underrepresented in global trial populations [32, 33] and in pandemic preparedness, they offer rapid, region-specific immunological assessment capability directly addressing the vulnerabilities exposed by COVID-19 [22].

Realizing this potential requires coordinated action. African governments must commit to STISA-2034 targets through dedicated R&D budget allocations and enabling policy frameworks [21]. African scientists must champion frugal, context-aware innovation, prioritizing local utility [42, 43]. International partners must transition from extractive toward genuinely equitable partnerships characterized by co-authorship, shared intellectual property, and African-governed data stewardship [48]. Implemented through the phased roadmap articulated herein, grounded in ethical governance and sustained political commitment, immune organoid platforms can position Africa not merely as a beneficiary of 21st-century biomedical innovation but as one of its defining contributors [21, 22].

## Data Availability

No datasets were generated or analysed during the current study.

## Abbreviations

DALY: Disability-adjusted life-year
GWAS: Genome-wide association study
hPSC: Human pluripotent stem cell
PDO: Patient-derived organoid
TB: Tuberculosis
AMR: Antimicrobial resistance
STISA-2034: Science, Technology and Innovation Strategy for Africa 2034
FRC: Fibroblastic reticular cell
AMRH: African Medicines Regulatory Harmonization
